# Mycoplasmotic Giant Cell Epitheliomatous Inverted Papillary Carcinoma of the Aural Canal

**DOI:** 10.4103/0974-777X.68548

**Published:** 2010

**Authors:** Mohammed Naim, Vanesa T. John, Amit Kumar, Khalid Iqbal

**Affiliations:** *Departments of Pathology and ENT, JNMC, AMU, Aligarh, UP, India*

Sir,

Recently, a report of mycotic giant cell epitheliomatous inverted papilloma appeared in your journal.[1] We present here the histopathological evidence of a mycoplasmotic giant cell epitheliomatous inverted papillary carcinoma.

A 45-year-old female with history of chronic asthma, chest X-ray report of interstitial changes in lungs, high resolution computerized tomography (HRCT) thorax report of soft tissue density lesions in the lung presented with ear problem, clinically diagnosed as “right aural polyp.” A small biopsy tissue from the polyp was submitted for histopathological diagnosis.

The biopsy section [Figure 1] revealed that the thickness of the keratinocytic strata decreased and hyperchromasia increased from right to the left (A). On the right plank, marked by intense eosinophil cell infiltrate, the basal layer presented discrete foci of hyperchromatic basal cells showing surface attached-elongate mycoplasma and protoplastic inclusions in the nucleus and cytoplasm (B). Occasionally, the basal cell displayed enlarged, intensely hyperchromatic, cleaved nucleus impending cell proliferation (Inset C). Parabasal epitheliomatous proliferation in the ret-e-pegs formed inverted papillae without a keratinocytic core (C). The infiltrate in the area became lymphocytic. Papillary cells divided by fission (Inset D left), became highly pleomorphic, occasionally showing burst, releasing mycoplasma-spheroplasts of 2–3 micron size (Inset D center) tending to elongate and reinfest (Insets D). Inverted papillae on the left displayed carcinoma cells dotted by giant cells also showing fission and burst releasing spheroplasts (Insets E).

**Figure 1:**
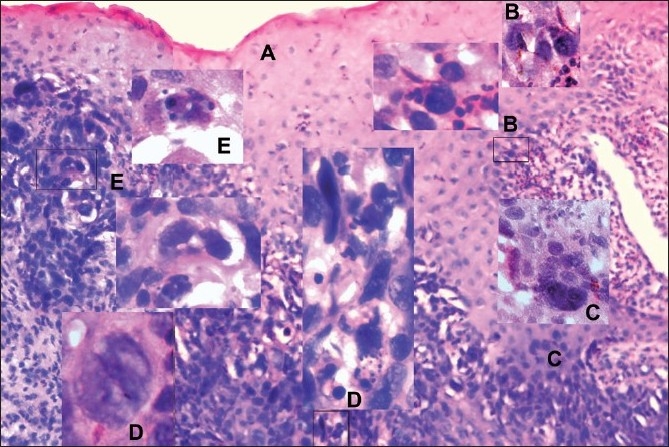
Evidences from the section stained with hematoxilin and eosin (H & E, ×125); insets B, C, D, E (H & E, ×1250)

Under high-power lens, the periodic acid Schiff (PAS)-stained section through deep invading papillae showed PAS-positive inclusions suspicious of histoplasma [Figure 2]. Oil immersion lens revealed PAS+ve replicate mycoplasma in the intercellular clefts, cytoplasm and nucleus of the infested and disrupted carcinoma cells (Inset A), showing protoplastic spheroid, oval, coccoid, balloon, disc, ring, star, granule and filament forms. Nuclear inclusions merged with the host cell chromatin inducing protoplastic character of forming nourishment vacuoles (Inset B). The inclusion-laden cytoplasm depicted (amoeboid) pseudopodia. Hybrid chromatin with its vacuole increased at the expense of infested-cell cytoplasm (inset C). The vacuole disappeared with increase of chromatin transformed into protoplastic (mycoplasmoid) spheroids of 1–2 microns, giving rise to mega mononucleate giant cells (inset D). The nuclear protospheroids enlarged, cleaving the nuclear membrane, to get released into the cytoplasm (inset E), giving rise to multinucleate giant cell awaiting fission.

**Figure 2:**
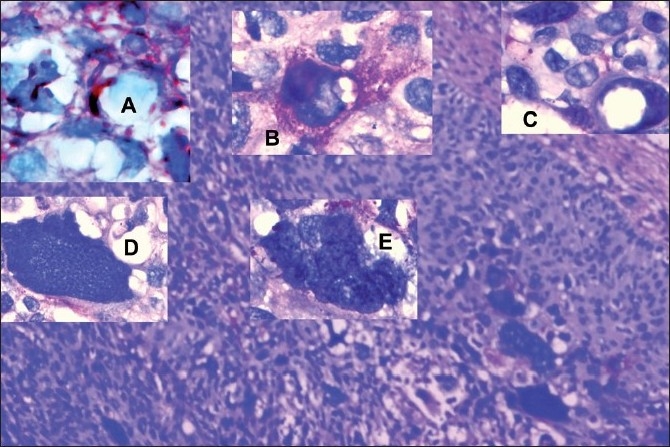
Evidences from sections stained with periodic acid Schiff stain (PAS, ×500); insets A, B, C, D, E (PAS, ×1250)

Histopathological evidence in this case was diagnostic of mycoplasmotic giant cell epitheliomatous inverted papillary carcinoma of the aural canal. Pneumonic asthma with otitis-media[2] and severe mucocutaneous lesions[3] were the known manifestations of mycoplasma, besides cancer signalling.[4] Presently, an immune-competent patient with pneumonic asthma presented aural polyp showing histopathological evidence of mycoplasmotic protoplastic transformation of the nuclear genetic content of the basal epithelial cells, resulting in carcinogenesis. Repetitive infestation of the cultured mouse embryo cells by mycoplasma was known to give rise to karyogenomic alterations and oncogenesis.[5] However, mycoplasma infestation of the basal cells with consequent protoplastic transformation of host cell nuclear chromatin and cell proliferation leading to epitheliomatous inverted papillary giant cell carcinoma in human beings, as observed presently, was not reported in the preexisting literature.

Mycoplasma, a self-replicating protoplastic microbe, lacking cell wall or membrane, having confluent cytoplasm and nuclear material, varying in size from microgranule to few microns, is identifiable by pleomorphic, granule, ovoid, spheroid and filament forms and PAS positivity. Mammalian cells are known for exerting positive taxis on mycoplasma due to which the organism stretches to elongate and attaches to the host mammalian cell, causing membrane damage, and gets phagocytosed across the host cell membrane barriers.[6] Histopathological evidence of basophilic intranuclear inclusions up to 15 micron and eosinophilic intracytoplasmic inclusions up to 8 micron in size were reported in epithelial cells of animals suffering from mycoplasmosis.[7] Presently, we observed PAS-positive inclusions of mycoplasma measuring few microns in the cytoplasmic as well as nuclear compartments of the freshly infested epitheliomatous carcinoma cells and consuming the host nuclear and cytoplasmic content by forming vacuoles. In further course, PAS-positive inclusions disappeared in the host nuclear chromatin, transforming it into PAS-negative, basophilic, protoplastic (mycoplasmoid) nucleic spheroids, suggestive of genomic hybridization, thus, transforming host cells into pleomorphic, protoplastic, giant, carcinoma cells. Keratinocytes were lately and lesser affected either due to the protective effect of saliva or some unknown biological factor.

Mycoplasma may parasitize and replicate in the basal epithelial cells, get released and reparasitize, leading to genomic alteration of the host cell and carcinogenesis of the giant cell epitheliomatous inverted papillary carcinoma. Keratinocytes are lately and less affected.

Protoplastic (protozooid) mycoplasma-replicate-inclusions and nuclear and cytoplasmic changes in the host cells constitute diagnostic histopathological features of the diseases.

Giant cells in the epitheliomatous carcinoma invite higher magnification histopathological examination for evidence of pathogenic organisms and infective carcinogenesis.

## References

[1] Naim M, Kumar A, John VT, Ahmad SS (2010). Mycotic giant cell epitheliomatous inverted papilloma of the gingiva. J Glob Infect Dis.

[2] Lambert HP (1969). Infections caused by Mycoplasma pneumonia. Br J Dis Chest.

[3] Sendi P, Graber P, Lepere F, Schiller P, Zimmerli W (2008). Mycoplasma pneumonia infection complicated by severe mucocutaneous lesions. Lancet Infect Dis.

[4] Gong M, Meng L, Jiang B, Zhang J, Yang H, WU J (2008). p37 from Mycoplasma hyorhinis promotes cancer cell invasiveness of MMP-2 and followed by phophorylation of EGFR. Mol Cancer Thr.

[5] Tsai S, Wear DJ, Shih JW, Lo SC (1995). Mycoplasmas and onchogenesis: infection and multistage malignant transformation. Proc Natl Acad Sci U S A.

[6] Zucker-Franklin D, Davidson M, Thomas L (1966). The interaction of mycoplasms with mammalian cells: 1.H_E_LA Cells, neutrophils, eosinophils. J Exp Med.

[7] Mitchell M Infectious diseases of captive reptiles (proceedings)-Veterinary Health Care. http://www.Vaternarycalendar.dvm.com.

